# *In Vitro* Anti-*Candida* Activity of the Hydroalcoholic Extracts of *Heracleum persicum* Fruit Against Phatogenic *Candida* Species

**DOI:** 10.5812/jjm.8703

**Published:** 2014-01-01

**Authors:** Batool Sadeghi Nejad, Mahsa Rajabi, Ali Zarei Mamoudabadi, Majid Zarrin

**Affiliations:** 1 Department of Medical Mycology, Medical School, Ahvaz Jundishapur University of Medical Sciences, Ahvaz, IR Iran; 2 Health Research Institute, Infectious and Tropical Diseases Research Centre, Ahvaz Jundishapur University of Medical Sciences, Ahvaz, IR Iran

**Keywords:** *Heracleum*, Extract, *Candida*

## Abstract

**Background:**

Nowadays *Candida albicans* has become resistant to the toxic and expensive commercial anti-Candida drugs. Therefore, investigation for new anti-fungal agents is necessary.

**Objectives:**

The purpose of this survey was to investigate the *in vitro* anti-*Candida* activity of the hydroalcoholic extracts of *Heracleum persicum* fruit.

**Materials and Methods:**

The plant ingredients were extracted using 80% ethanol and the extract was screened against 46 isolated pathogenic *Candida* species such as *C. albicans*, *C. glabrata* and *C. tropicalis* by agar well diffusion method.

**Results:**

The minimum inhibitory concentration (MIC) values at 24 and 48 hours were 0.625 - 20 µg/µL for *C. albicans*, 0.625 - 40 µg/µL for *C. glabrata*, and 5.0 - 20 µg/µL for *C. tropicalis*.

**Conclusions:**

The results of this survey confirmed that tested plant extract had a potential anti-*Candida* activity. Hence, it is suggested to isolate and identify its active compounds in future.

## 1. Background

In the past two decades, the prevalence of candidiasis has been increased. *Candida* species are able to create superficial and systemic infections. *Candida albicans* is an opportunistic pathogen, causing mycoses in immunocompromised patients as well as long-term antibiotic users (1). Also, other *Candida* species such as *C. glabrata*, *C. parapsilosis*, *C. tropicalis *and *C. krusei* are among the oral mucosal lesions suspected agents in AIDS patients (2). Herbal Medicines have been used as alternative drugs in developing countries. Brazil, Cuba, India, Jordan and Mexico are examples of countries with various herbs as well as a potent folklore in using medicinal plants for their antimicrobial and antifungal benefits (3-6). *Heracleum persicum* (in the *Apiaceae* family), known as Golpar in Persian, is vernacular to Iran. It grows wildly in humid mountainous regions and is used in soups and stews. Ten out of 70 species (7) of *H. persicum* are known in Iran (8). *H. persicum* fruit is extensively used as spice and the young stems are also used for making pickles. Chemical compounds such as Pimpinellin, isopimpinellin, bergapten, isobergapten and six furanocoumarins have been reported to be extracted from its roots (9, 10).

## 2. Objectives

Because of the high usage of *H. persicum* fruit as an herbal medicine in the Iranian culture beside its analgesic activity, we decided to survey the hydroalcoholic extract of *H. persicum* fruit, assessing its anti-*Candida* activity by agar well diffusion method.

## 3. Materials and Methods

### 3.1. Plant Ingridients and Preparation of Hydroalcoholic Extract

*H. persicum* fruits were purchased from a local market in Ahvaz, Iran. For preparation of hydroalcoholic extract, 10 g air-dried and powdered fruit of *H. persicum* was macerated with 100 mL of 80% ethanol and methanol on a rotary shaker for 72 hours, filtered, and then the solution was evaporated in the room temperature. Dried hydroalcoholic extract was stored in a sterile glass bottle at -20°C until future assays. One gram of the dried hydroalcoholic extract of *H. persicum* was dissolved in 5 mL dimthyl sulphoxide (DMSO, 100%) to a final concentration of 200 µg/µL as stock, and serial double fold dilutions were prepared using sterile distilled water from 0.078 - 40 µg/µL according to the previous literature (11).

### 3.2. Yeast Inoculum Preparation

A total of 47 *Candida* spp. isolates including *C. albicans* [29], *C. glabrata* [10], and *C. tropicalis* [7], isolated from oral swabs samples, were selected.* Candida* spp. isolates were inoculated into Sabouraud dextrose broth (SDB, Merk, Germany) and grown overnight on a rotary shaker at room temperature. Then cells were washed three times with sterile distilled water and adjusted by the same solvent to yeast inoculum of 10^6^ CFU/mL (0.5 Mac-Farland standard).

### 3.3. Positive and Negative Controls

The commercial antifungal drugs such as clotrimazole disc (10 µg/disc) and nystatin disc (100 IU/disc) were used as positive controls and DMSO was used as negative control.

### 3.4. Anti-Candida Assay

Anti-*Candida* activities of the hydroalcoholic extracts of *H. persicum* fruit were assayed against *Candida* spp. isolates by agar well diffusion according to Perez et al. method (12). One hundred microliter of yeast inoculum (10^6^ cells/mL) was uniformly spread onto Sabouraud dextrose agar medium (SDA, Merck, Germany) using a bent glass rod. Then five wells of 7 mm diameter were punched by a borer into the SDA medium and filled with 100 µL of two-fold serial dilutions of plant extracts as well as sterile DMSO 100% as negative control. Plates were incubated for 24 hours at 37°C. Anti-*Candida* activity was determined by measuring the zone of inhibition. Experiments were carried out three times. Two positive controls such as clotrimazole and nystatin discs were placed in the plate. The lowest concentration of a tested plant extract exhibiting a clear zone, was considered as the minimum inhibitory concentration (MIC).

## 4. Results and Discussion

**Figure 1.**
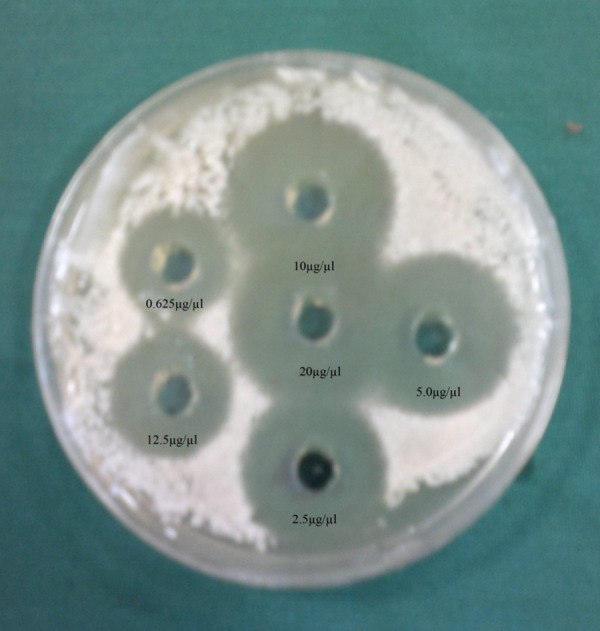
Anti-*Candida* Activity Assay of the Ethanolic Extract of *H. persicum *Fruit Against *C. albicans *Using Agar Well Diffusion Method Decreasing dilutions ranging from 0.625 to 20 µg/µL; MIC = 0.625 µg/µL.

In the current study, we surveyed the anti-*Candida* activity of the hydroalcoholic extracts of *H. persicum *fruit against three different *Candida *species (*C. albicans *, *C. tropicalis *and *C. glabrata*). The results are summarized in Figure 1 and Tables 1 and 2. The ethanolic and methanolic extracts of the tested plants showed anti-*Candida* activities against *C. albicans *(n = 29), *C. tropicalis *(n = 10), and *C. glabrata *(n = 7). The strongest activity was seen against *C. albicans *with a range of 12 - 21 mm inhibition zones and 0.625 - 20 µg/µL MIC values; the ethanolic extract of the tested plant has more anti-*Candida* effects at 0.625 µg/µL compared to the methanolic extract at 2.5 µg/µL. Similar results were reported by other researchers with different medicinal plants (13). According to Mimica-Dukic et al. study (14) among tested *Candida *spp. isolates, *C. albicans *was the most sensitive tested *Candida *spp. to *Mentha piperita *L. oil, which was in agreement with our results. The lowest concentration of the tested plant showed a potential anti-*Candida *activity against *C. albicans *and *C. glabrata *, while the highest concentration showed a weak inhibitory effect against *C. tropicais *. Furthermore, previous studies reported that the essential oil of *H. persicum *has moderate anti-*Candida* activity (15). In Iranian traditional medicine, *H. persicum *fruit was used as a carminative and pain relieving herbal drug ( 16 , 17 ). Review of literature reported that coumarins were known to be responsible for antifungal activity of many of the medicinal plants (18, 19). It was suggested that furanocoumarins, isolated from the fruit of *H. persicum *(10), were the cause of anti-*Candida* activity of this plant. *Candida *species are in the normal flora of healthy people, while they can cause superficial mycosis and invasive infections in immunocompromised patients (20). During the past 20 years, the incidence of infection by pathogenic fungi has been increased; most of which were infected by *Candida *spp. that can change the superficial mycosis to invasive infections. *C. albicans *has been a major factor of morbidity and mortality in immunocompromised patients, but other *Candida *spp. such as *C. glabrata *and *C. krusei *have expanded in the recent years (21 - 23). 

**Table 1.** The MIC Values of the Hydroalcoholic Extracts of *H. persicum *Fruit Against *Candida *spp. Isolates

Antifungal Agents	*Candida* spp. Isolates MIC ^a^, µg/µL		
	*C. albicans*, No. 29	*C. glabrata*, No. 10	*C. tropicalis*, No. 7
**Treatments^b^**			
Ethanolic**extract of *H. persicum*	0.625 - 20	0.625 - 40	5 - 20
Methanolic extract of *H. persicum*	5 - 20	2.5 - 20	5 - 20
**Positive controls**			
Clotrimazole	0.0078 µg/µL	-- ^c^	--
Nysatatin	0.0039 µg/µL	--	--

^a^ Abbreviation: MIC, minimal inhibitory concentration.

^b^ Tests were done in triplicate. Tested concentrations: extracts, 40 µg/µL; positive controls, Clotrimazole 2 µg per well and Nysatatin 2 µg per well.

^c^ no data is provided.

**Table 2.** The Zone of Inhibition of the Hydroalcoholic Extracts of *H. persicum *Fruit Against *Candida *spp. Isolates

Antifungal Agents	*Candida* spp. Isolates Inhibition Zone Diameter, mm		
	*C. albicans*, No. 29	*C. glabrata*, No. 10	*C. tropicalis*, No. 7
**Treatments** ^a^			
Ethanolic extract of *H. persicum*	10 - 18	11 - 18	8 - 15
Methanolic extract of *H. persicum*	12 - 21	12 - 20	11 - 18
**Positive controls**			
Clotrimazole	20	-- ^b^	--
Nysatatin	22	--	--

^a^ Tests were done in triplicate. Tested concentrations: extracts, 40 µg/µL; positive controls, Clotrimazole 10 µg/disc and Nysatatin 100 IU/disc.

^b^ no data is provided.

Therefore, there is an urgent necessity for finding alternative antifungal drugs for effective treatment of Candidal infections.
